# In Vitro Cultures of Adipose-Derived Stem Cells: An Overview of Methods, Molecular Analyses, and Clinical Applications

**DOI:** 10.3390/cells9081783

**Published:** 2020-07-27

**Authors:** Maurycy Jankowski, Claudia Dompe, Rafał Sibiak, Grzegorz Wąsiatycz, Paul Mozdziak, Jędrzej M. Jaśkowski, Paweł Antosik, Bartosz Kempisty, Marta Dyszkiewicz-Konwińska

**Affiliations:** 1 Department of Anatomy, Poznan University of Medical Sciences, 60-781 Poznan, Poland; mjankowski@ump.edu.pl (M.J.); 75094@student.ump.edu.pl (R.S.); m.dyszkiewicz@ump.edu.pl (M.D.-K.); 2 Department of Histology and Embryology, Poznan University of Medical Sciences, 60-781 Poznan, Poland; claudia.dompe.16@abdn.ac.uk; 3 The School of Medicine, Medical Sciences and Nutrition, Aberdeen University, Aberdeen AB25 2ZD, UK; 4 Department of Veterinary Surgery, Institute of Veterinary Medicine, Nicolaus Copernicus University in Torun, 87-100 Toruń, Poland; g.wasiatycz@umk.pl (G.W.); pantosik@umk.pl (P.A.); 5 Physiology Graduate Program, North Carolina State University, Raleigh, NC 27695, USA; pemozdzi@ncsu.edu; 6 Department of Diagnostics and Clinical Sciences, Institute of Veterinary Medicine, Nicolaus Copernicus University in Torun, 87-100 Toruń, Poland; jmjaskowski@umk.pl; 7 Department of Obstetrics and Gynecology, University Hospital and Masaryk University, 20 Jihlavská St., 601 77 Brno, Czech Republic; 8 Department of Biomaterials and Experimental Dentistry, Poznan University of Medical Sciences, 60-812 Poznan, Poland

**Keywords:** adipose-derived stem cells, clinical trials, molecular studies, in vitro

## Abstract

Adipose-derived stem cells (ASCs) exhibiting mesenchymal stem cell (MSC) characteristics, have been extensively studied in recent years. Because they have been shown to differentiate into lineages such as osteogenic, chondrogenic, neurogenic or myogenic, the focus of most of the current research concerns either their potential to replace bone marrow as a readily available and abundant source of MSCs, or to employ them in regenerative and reconstructive medicine. There is close to consensus regarding the methodology used for ASC isolation and culture, whereas a number of molecular analyses implicates them in potential therapies of a number of pathologies. When it comes to clinical application, there is a range of examples of animal trials and clinical studies employing ASCs, further emphasizing the advancement of studies leading to their more widespread use. Nevertheless, in vitro studies will most likely continue to play a significant role in ASC studies, both providing the molecular knowledge of their ex vivo properties and possibly serving as an important step in purification and application of those cells in a clinical setting. Therefore, it is important to consider current methods of ASC isolation, culture, and processing. Furthermore, molecular analyses and cell surface properties of ASCs are essential for animal studies, clinical studies, and therapeutic applications of the MSC properties.

## 1. Introduction

Adipose-derived stem cells (ASCs) are a population of multipotent, plastic adherent cells obtained through collagenase digestion of white adipose tissue [1]. Exhibiting mesenchymal stem cell (MSC) characteristics, ASCs have been extensively studied in recent years. ASCs have been reported to exhibit high stemness, differentiating into lineages such as osteogenic, chondrogenic, neurogenic, or myogenic [2]. The particular focus of most of the current research concerns either their potential to replace bone marrow as a readily available and abundant source of MSCs, or to employ these stem cells in regenerative and reconstructive medicine [3]. The studies of MSCs derived from adipose tissue have been especially successful, identifying it as an abundant and easily accessible source of these cells. Furthermore, currently, there is a growing number of clinical approaches involving ASCs, which emphasizes the need for research that analyzes the molecular bases of their functioning, both in vivo and in vitro, as well as their possible interaction with cells or tissues in the organism of the patients [4,5,6]. A vast number of such studies, at some stage, employ in vitro cultures for ASC propagation, differentiation, as well as collection of material for subsequent use in both molecular analyses and in vivo applications [7]. Hence, despite the constant advancements concerning the clinical use of ASCs, in vitro studies continue to be relevant, providing the necessary cellular and molecular reference needed to enable future widespread application of therapies based on these cells.

Therefore, the focus of this review is placed on in vitro studies of ASCs, presenting the current methods of their isolation, culture, and processing. Furthermore, it summarizes the molecular analyses of these cells, as well as presents the currently available animal and clinical studies taking advantage of their MSC properties. All of the publications cited in the manuscript were sourced from reputable scientific databases, such as PubMed or Scopus, with inclusion in the review warranted by their overall recency, credibility, and comprehensiveness.

## 2. Methods of Isolation, Culture, and Processing

Initially, ASC studies sourced these cells from adipose tissue fragments obtained through or during surgery. In this approach, the obtained fragments require mincing into very fine fragments, usually with the use of surgical blades [3]. Then, the minced tissue is subjected to extensive washing, to remove any traces of hematopoietic stem cells. Furthermore, the tissues are subjected to collagenase digestion to achieve their full cellular liberation [8]. Cell suspension obtained in this manner is centrifuged, allowing for separation of mature adipocytes in the upper fraction from the stromal vascular fraction pellet [9]. The cell mix remains heterogenous, containing peripheral blood mononuclear cells, fibroblasts, and epithelial cells, in addition to the ASCs. However, subjecting the cells to a plastic adherence assay and allowing them to attach to culture plates for 72 h, permits selection of the population of adipocyte precursors present in the fraction [10].

**Figure 1:**
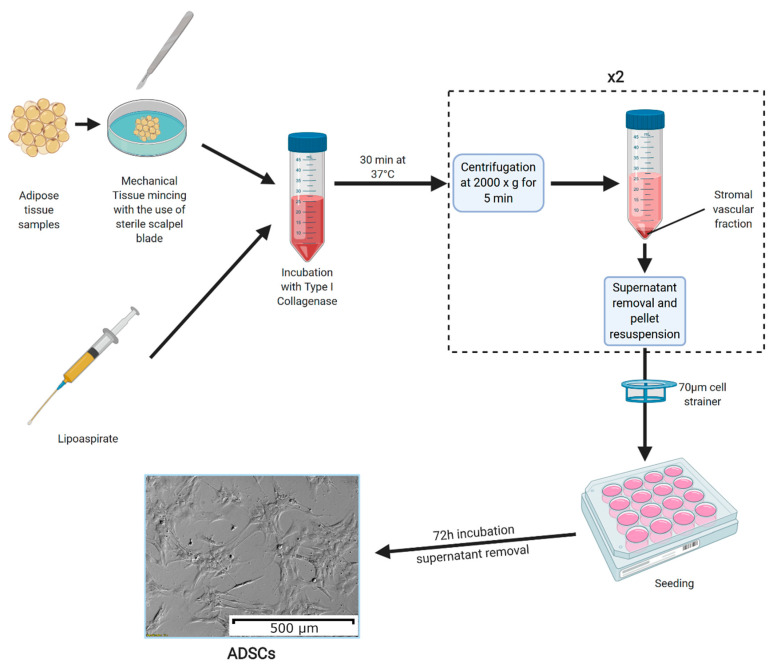
The methods of adipose-derived stem cell (ASC) culture preparation. Adipose tissue samples are mechanically minced into small fragments (the process is not needed if ASCs are obtained from lipoaspirates). Then, the tissue fragments are incubated in a solution of type I collagenase for 30 min at 37 °C. The collagenase is neutralized using a 5% fetal bovine serum solution in culture medium, with the samples centrifuged afterwards at 2000× *g* for 5 min. Furthermore, the resulting supernatant is discarded, and the pellet is resuspended in culture medium, after which the centrifugation step is repeated. The obtained pellet is again resuspended and filtered through a 70 µm cell strainer. The resulting cell suspension is seeded onto culture plates and left to adhere for 72 h. After this time, the culture medium is removed, leaving ASCs adhered to the plate bottom [13]. The photograph of ASC morphology was taken using an inverted microscope with 10× magnification lens. Created with Biorender.

In modern studies, because liposuction surgery is performed increasingly more often around the world, ASCs are usually sourced from the obtained lipoaspirate, which does not require further mechanical processing, as the procedure produces a saline suspension of very small tissue fragments [11]. Furthermore, studies investigating the viability of the obtained ASCs found no significant difference between the cells obtained through both of the abovementioned methods [12]. The full process of culture preparation, most commonly described in the literature, is presented in Figure 1.

**Figure 2:**
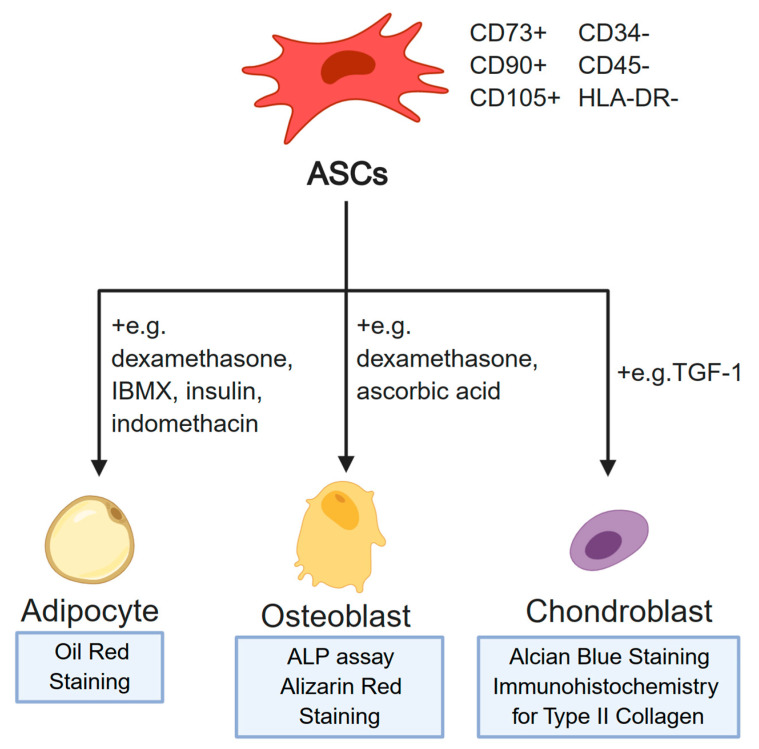
The minimal criteria necessary for confirmation of the mesenchymal stem cell (MSC) phenotype of ASCs. The minimal set of markers is presented topmost. Examples of media supplements used in differentiation into specific cell lineages are indicated next to the lines representing the differentiation process. Furthermore, the widely accepted assays for confirmation of the identity of each differentiated cell population are provided at the bottom of the figure. Created with BioRender.

ASCs obtained through the abovementioned methods can be further identified using two basic approaches. Firstly, cell surface proteins that are characteristic for this MSC population can be detected through methods such as flow cytometry. The available literature describes the minimal set of markers necessary for positive identification of MSCs [14,15]. The cells need to be CD73, CD90, and CD105 positive, and at the same time not exhibiting the expression of CD34, CD45, and HLA-DR [16]. However, the final confirmation of the MSC phenotype of ASCs is their ability to differentiate into three characteristic lineages [14]. Firstly, upon the addition of factors such as dexamethasone and ascorbic acid, the cells should assume osteoblast phenotype [17]. Then, differentiated osteoblasts can be detected using ALP (alkaline phosphatase) assay or alizarin red staining [18]. Furthermore, the addition of TGF-β1 stimulates ASC differentiation towards the chondrogenic lineage [19]. In this case, the successful lineage commitment can be detected using either alcian blue staining, or immunocytochemistry targeted at detecting type II collagen formations in the cells [20]. Finally, adipocyte differentiation is achieved through addition of factors such as dexamethasone, IBMX, insulin, and indomethacin to the culture medium, with the resulting cells detectable using Oil Red staining [21]. The complete minimal criteria for characterization of MSC characteristics of ASCs are presented in Figure 2.

**Figure 3:**
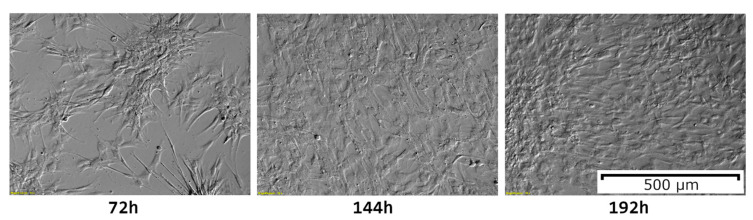
ASC morphological changes over 192 h of primary culture. The initial shape of the cells can be observed to change due to culture density. In the 192 h of the culture, 95% confluence can be observed, indicating readiness for passaging or collection. The photographs included in the figure were taken using an inverted microscope at 10× magnification.

ASCs can be relatively easily maintained in culture, ready to be passaged or harvested after around 192 h [22]. FBS (fetal bovine serum) is the most commonly used serum supplement of such cultures. However, some sources suggest alternative sources of growth factors for the ASCs [23]. Human platelet cell lysate addition causes a significant increase in cell proliferation as compared with FBS, and it has been shown to cause some gene expression changes, which could have some influence on the overall properties of ASCs [24,25]. In turn, when allogenic human serum was also examined as a supplement, it was shown to be slightly less potent, requiring higher concentrations than FBS to achieve the same effect [26]. Most of the sources agree that the cells should be harvested at the confluence of 90–95%, as cultures of excessive density can affect their gene expression [13,27]. Density dependent changes in ASC morphology are presented in Figure 3.

After detachment of the cells from the culture vessels using trypsin digestion, the samples can be subjected to molecular analyses, processed for nucleic acid or protein isolation, or frozen for further use [13]. For the latter purpose, freezing in 10% solution of DMSO in, for example, human serum albumin, shows satisfactory results [28]. Some studies have reported that following a specific procedure of freezing and thawing could improve cell viability after freezing, improving reliability of frozen MSC stocks [29].

Overall, the methodology of ASC isolation, culture, identification, and processing is thoroughly described in the literature with close to consensus. This factor, as well as the accessibility and relative of collection of adipose-derived MSCs, in vitro cultures of these cells have a large potential to serve as a basis for both molecular and potential clinical studies.

## 3. Molecular Analyses of In Vitro Cultured ASCs

### 3.1. Osteogenic Differentiation

ASCs, due to their multilineage differentiation ability, have great potential in tissue regeneration, including bone reconstruction. However, a deeper understanding of the molecular mechanisms underlying ASC osteoblastic differentiation could lead to novel applications treating a multitude of different bone-related diseases. Multiple factors influence the commitment of ASCs and their differentiation to the osteolineage. For instance, the parathyroid hormone, PTH1-34, orchestrates bone formation influencing osteo-induced ASCs by phosphorylating SIK2, upregulating RANKL and Wnt4, and downregulating SOST. Wnt4 knockdown inhibits osteogenic differentiation altering the expression of downstream osteogenic proteins. These results indicate that further understanding of PTH1-34 controlling SIK2 and Wnt4 signaling pathways and their role in ASC osteogenesis could provide new applications for bone regeneration [30]. Moreover, Notch proteins which are a family of key regulator ligands involved in osteogenesis, were observed to also impact on ASC proliferation and differentiation. These proteins were previously described as bone-marrow derived stem cells (BMDSCs) osseous differentiation regulators. Similarly, the inhibition of Notch and the associated downregulation of ASC proliferation and osteoinduction has been found to be a useful potential translatable “on/off switch” in the regulation of proliferation, differentiation, and osteogenic potential of ASCs. Additionally, delivery of Notch-1 intracellular domain (NICD), after prior Notch inhibition, restored bone formation [31].

Evaluation of the osteogenic marker expression in ASC growth under two-dimensional (2D) and three-dimensional (3D) cell culture conditions has been compared to analyze the influence of the extracellular matrix (ECM) structure. The osteogenic marker, CBFA-1, was quantified through real-time PCR and it was upregulated in both cultures. However, cells seeded on 3D cell culture, showed faster growth, and also greater expression of CBFA-1 and other osteogenic markers, suggesting that 3D cultures overstimulate osteogenic differentiation of ASCs as compared with 2D culture [32]. Because stem cells which usually reside in a multifactorial environment, with different biochemical and mechanical signals affecting their properties, are subjected to various and continuous changes, elucidating the mechanisms underlying niche cues and the responses connected to them is complicated. Most studies on niche interactions have been carried out on 2D surfaces, which is not a truthful representation of their natural 3D environment [33]. In fact, in 3D environments, multiple factors interplay and cannot be controlled, consistently differing from 2D cultures [34]. Three-dimensional combinatorial hydrogels with independent control of biochemical and mechanical properties have been designed to analyze signals of the niche on stem cells osteogenesis in in vitro culture. This scaffold promoted bone differentiation at specific combinations, leading to low fibronectin and high osteocalcin gene expression. Enough support for the conduction of mechanistic studies has been provided in order to elucidate niche cues regulating stem cell fate and identify the best niche cue promoting the desired differentiation pattern [35].

Finally, studies focusing on miRNAs’ functions have examined their roles in the osteogenesis of ASCs and the key genes involved in the process [36,37]. Gene ontology and pathway analysis have been performed and a network of 72 mRNAs and 9 miRNAs were predicted to be involved in controlling osteogenic differentiation of ASCs. Six of these miRNAs (miR-143-3p, miR-135a-5p, miR-31-5p, miR-22-3p, miR-193b-3p, and let-7i-5p) were found to be strictly related with osteogenic differentiation of ASCs and a novel regulator of osteogenesis of ASCs, DPYSL3, was identified, unravelling novel applications of ASCs in bone regeneration-associated disease [38].

### 3.2. Neurogenic Differentiation

ASCs’ differentiation ability towards neuron-like cells shows great potential in therapies treating diseases of the nervous system. The mechanisms underlying the in vitro differentiation of ASCs into immature neuron-like cells is similar to adult neurogenesis. In fact, ASC differentiated cells show similar neural markers expression to neural cells, including the proneural factors, such as Pax6, Mash1, Ngn2, NeuroD1, Tbr2, and Tbr1, and their pattern of expression is the same as in the intermediate stages of neuronal differentiation [39]. Moreover, the application of ASCs in the treatment of peripheral nerve injuries is possible due to their plasticity towards Schwann cells (SC). In fact, the ASCs from rat visceral fat, following treatment with glial growth factors, adopted a spindle-like morphology typical of SCs expressing of glial markers [40]. Following in vitro stimulation with growth factors, the neurotrophic and angiogenic properties of hASC, and their involvement in peripheral nerve injury model, were evaluated. Increased expression of neurotrophic and angiogenic molecules, as well as a rapid axon regeneration and angiogenesis, were observed. Moreover, the secretion of brain-derived neurotrophic factor (BDNF), glial cell-derived neurotrophic factor (GDNF), vascular endothelial growth factor-A (VEGF-A), and angiopoietin-1 proteins were enhanced. The conditioned medium from stimulated cells promoted the outgrowth of dorsal root ganglia (DRG) neurons, and c-jun and caspase-3 expression was reduced in the latter [41]. ASCs from mice, induced for neurogenesis, showed neuron-like morphology and expressed neural markers, including glial fibrillary acidic protein, nestin, MAP2, and β-tubulin III. Evidence proved that ghrelin concentration increased the proportion of neural-like cells, branching dendrites, and the expression of neural markers. Ghrelin was demonstrated to promote neurogenesis activating β-catenin and AKT/mTOR signaling pathways, important for cell growth, survival proliferation, angiogenesis, translation, transcription, and metabolism, whose inhibition suppressed ghrelin-induced neurogenesis [42].

A study that investigated the effects of ECM molecules on proliferation, adhesion, and cell viability of SC-like differentiated ASC showed it increased the neurotrophic potential of stem cells. When exposed to apoptotic conditions, two key molecules, fibronectin and laminin, increased the viability and the adhesion of ASCs but had no effect on proliferation. Neurite outgrowth of DRG neurons was enhanced both when they were in direct contact with ASCs and when the latter were seeded on laminin and fibronectin, while they did not affect growth factor levels nor the secretion from ASC of brain derived neurotrophic factor. Overall, ECM molecules increased ASCs’ potentiality in nerve regeneration [43].

Finally, a study analyzed the influence of different induction times on proliferation, differentiation, and secretion abilities of ASC-induced SC-like cells. According to the results, different induction times negatively impacted proliferation but positively impacted the expression of SCs. Application of induced SCs for nerve repair and functional reconstruction upon nerve injury appeared to be the most beneficial after 19 days of induction [44].

### 3.3. Role in Metabolism

Proteome analysis of primary cultures of ASCs has been carried out to further understand adipogenesis, especially in relation to energy metabolism and the etiology of obesity, and showed altered expression of protein typical of metabolism, redox, protein degradation, and heat shock protein/chaperones. Additional analysis correlated the induction of heat shock proteins with a possible role of ASCs in obesity and type 2 diabetes, indicating a need for further research [45]. The adipose tissue has often been referred to as an endocrine organ as multiple adipokines released by adipocytes, including adiponectin and vaspin, have hormone-like activities. An in vitro study, analyzing the molecules secreted by subcutaneous ASCs, reported elevated expression of actin and lactate dehydrogenase and a wide range of adipokines, including adiponectin and multiple serpins, additionally, suggesting a possible role of ASCs in the development of obesity and type 2 diabetes [46].

Subcutaneous adipose tissue and visceral adipose tissue, responsible for metabolic diseases defense, are involved in fat tissue homeostasis. Although their differentiation, proliferation, and adipogenic potentials are fundamental for this process, by promoting adipocyte hyperplasia and limiting disorders, the molecular pathways regulating ASCs in these two types fat tissues, and their relative metabolic properties, is not yet well understood. However, different functions of visceral and subcutaneous ASCs are thought to be regulated by the key protein CD90, often anchored to the glycosylphosphatidylinositol of cells playing a key role in proliferation, mitotic clonal expansion, and homeostasis of adipose tissue and metabolism. CD90 was differently expressed in visceral and subcutaneous ASCs and further analysis could lead to advances in the treatments of multiple metabolic disorders [47].

### 3.4. Cardiovascular Research

MSCs show great potential in the treatment of cardiac injury following myocardial loss, but the best source of MSCs and the optimal condition for the induction of in vitro cardiac differentiation is yet to be defined. ASCs present differentiation potential towards endothelial tissue and they are involved in angiogenesis and vasculogenesis, showing possible applications in the treatment of cardiovascular diseases. ASCs have the ability to differentiate towards cardiomyocytes and protect pre-existing cardiac cells through their paracrine activity, releasing antiapoptotic factors [48]. Moreover, differentiation of BM-MSCs and ASCs towards cardiomyocytes showed a similar marker profile and proliferations rate; however, the expression of cardiac specific markers was higher in TGF-β1 induced ASCs, proving this source of stem cells to be ideal for stem cell therapy in cardiovascular diseases [49].

### 3.5. Vaculogenesis and Endothelial Differentiation

In addition to their acclaimed osteogenic differentiation potential, ASCs’ plasticity can generate endothelial tissue, being involved in the process of angiogenesis. The ability of ASCs to promote capillary network development is involved in adipose tissue physiology which is required for tissue enlargement, and this property can be exploited for in vitro reconstruction of hard tissue, employing vasculogenic elements. The investigation of ASCs’ adhesion, distribution, proliferation, and gene expression showed osteogenic and vasculogenic commitment. The analysis of chromosomal stability showed no alteration in long-term in vitro cultures. Nonetheless, coculturing specific cells with endothelial cells increased vascularization which is a process often considered to be a burden in tissue engineered grafts, and ASCs’ co-commitment to osteogenic and endothelial cell lineages increased the expression of osteogenic markers, hence, osteogenesis was increased when its commitment co-occurred with the vasculogenic commitment [50].

### 3.6. Cancer Research

ASCs are widely studied in cancer progression and development. A study of ASCs from patients affected by ovarian cancer showed high expression levels of α-smooth muscle actin (α-SMA). Moreover, epithelial ovarian cancer cells (EOCCs) stimulated the expression of carcinoma-associated fibroblast (CAF)-like markers in ASCs, while the latter promoted the proliferation and progression of EOCCs. The results suggested that ASCs were a source of CAFs and that they influenced the interaction of EOCCs with the microenvironment of the adipose tissue [51]. Therefore, ASCs affect the growth and metastasis of ovarian cancer, but underlying mechanisms have not been fully understood yet. However, the protein expression of ovarian cancers cells has been compared with protein expression following treatment ASCs, revealing that thymosin beta 4 X-linked (TMSB4X) accelerated ASC-mediated proliferation, invasion, and migration of ovarian cancer cells [52].

ASCs were analyzed to better understand the mechanisms underlying upper limb lymphedema complications upon axillary surgery in breast cancer patients, through the study of the stromal fraction of lymphedema-associated fat and the adipogenic transformation. Osteogenic, adipogenic, and vasculogenic gene expressions were examined. Lymphedema-associated stem cells showed enhanced adipogenic expression and a high ability to differentiate into adipose tissue, but low vasculogenic gene expression and no difference in osteogenic differentiation potential. These results suggested that the pathophysiology of lymphedema promoted the adipogenic differentiation of ASCs [53].

### 3.7. Tissue Regeneration Research

Adipose tissue is accepted as a source of ASCs for regenerative medicine and tissue reconstruction. However, long lasting graft retention is not always successful. Transcriptome analysis of ASCs has been carried out to analyze the changes in cell functions during development into mature fat cells. Microarrays analysis of RNA from in vitro cultures of ASCs confirmed the expression of multiple genes associated with adipogenesis, such as the adipocyte-specific genes *FABP4*, *ADIPOQ*, and *PLIN4*, and it revealed numerous changes in the mRNA expression profile throughout the maturation process. For example, the expression pattern of *FGF11* suggested it influenced mature adipocyte phenotype maintenance. The expression of RSAD2 which is an interferon-inducible gene acting against multiple viral pathogens, suggested it could be synthesized together with depositories of accumulated lipids. Finally, both HES1 and periostin were inhibited. Although the downregulation of periostin still needed to be evaluated, lower expression of HES1 was associated with the maintenance of committed, but undifferentiated ASCs [54]. The regulation of transcription and metabolism of ASCs was further analyzed by treatment with fatty acid-binding proteins 4 (FABP4) and 5 (FABP5), and lipid chaperones expressed in adipocytes. FABP4 is secreted during lipolysis, and functions as an adipokine affecting genes associated with inflammatory and metabolic responses and influences cell differentiation. These proteins were observed to affect ASCs, thus, proving that the adiposity of the host environment influenced ASCs’ properties, and therefore impacted the range of possible applications in regenerative medicine [55]. An in vitro study aimed at analyzing the impact of articular microenvironment of rheumatoid arthritis (RA) on the therapeutic effects of ASCs. ASDCs’ response was altered upon treatment with synovial fluids from patients suffering from RA and coculture with macrophages or T-cells, further proving that the local environment influenced ASCs. Further analysis is required to clarify the immunomodulatory potential of ASCs and fully take advantage of their clinical benefit [56].

Moreover, the action of microRNAs, the regulatory activity they play on ASCs, and the ability they show to stimulate vascular network restoration, are beneficial for tissue repair; miR-92a is highly expressed in ASCs, and transfection of ASCs with pre-miR-92a or anti-miR-92a changed the expression of target genes. Elucidating paracrine mechanisms, genome, and secretome analysis of ASCs upon transfection with anti-miR-92a showed an increased expression of VEGF, angiogenin, and leptin and overexpression of miR-92a in ASCs showed a decreased secretion of hepatocyte growth factor (HGF) and angiopoetin-1. It is clear that miR-92a affects ASCs and the underlying mechanisms need to be further investigated [57].

Potential for novel therapeutic strategies of tissue repair can be found in the activity of exosomes derived from ASCs. These nanoscale vesicles of endocytic origin affect receptor cells influencing cell-to-cell communication. Proteomic analysis of exosomes originating from ASCs has revealed expression of proteins typical of cells responsible for protein binding, mostly participating in function prediction, posttranslational modification, and chaperoning. Some proteins detected are commonly involved in metabolic pathways, focal adhesion, regulation of the actin cytoskeleton, and microbial metabolism, together with tissue repair-related signaling pathways, such as putative paracrine effectors of angiogenesis including platelet-derived growth factor, epidermal growth factor, fibroblast growth factor, and nuclear factor kappa B (NF-κB) signaling pathway proteins [58].

### 3.8. Other Applications

Other applications of ASCs have focused on the treatment and prevention of dermis conditions. ASCs secrete soluble factors affecting skin biology in different ways, including protecting human dermal fibroblast from oxidative injury, through antioxidant and reduction activity in apoptotic cells. Proteomic analysis showed the activity of ascorbic acid activity on other antioxidant proteins, together with morphological changes, to increase resistance to free radicals, great advantage for skin damage prevention, and treatment of skin conditions [59]. ASCs from chyle were analyzed to identify their effects on hypertrophic scar (HS) formation, usually caused by an injury to deep layers of the dermis, characterized by excessive collagen deposition. These cells showed adipogenic and osteogenic differentiation potential and their use in the treatment of hypertrophic scar–derived fibroblasts changed cell proliferation, migration, and protein expression of scar-related molecules. Their paracrine activity suggested an inhibition of fibrosis [60]. In fact, ASCs release paracrine factor RNAs and extracellular proteins, including cytokines and growth factors, which promote healing and show therapeutic effects [61,62]. Moreover, ASCs also produce antioxidants, chaperone proteins, angiogenic, and antiapoptotic factors [1,6]. However, the secretory and signaling proteins, together with their multipotency potential and functions, change in the functionally heterogeneous population of stem cells contained by ASCs [62]. White and brown adipose tissues both secrete adipokines, including the hormones leptin and adiponectin, either directly or indirectly through vesicles such as exosomes [63]. Finally, a study aimed at determining if ASCs express phenotypic specific markers of keratocytes showed similar expression levels of differentiation markers to corneal stromal stem cells, suggesting potential for corneal cell therapy and tissue engineering [64].

In conclusion, ASCs have the potential to differentiate into multiple cell lineages and the analysis of the molecular pathways underlaying these processes could lead to a greater understanding of these mechanisms. Further research on this matter could expand the possible application of ASCs to a wide range of clinical therapies bringing advances to the treatment and diagnosis of multiple diseases.

## 4. Clinical Applications, Current and Perspectives

### 4.1. Animal Models

Animal models play a key role in the understanding of the biological activity of ASCs in a range of human diseases and disorders. Animal models allow the histological assessment of changes in the examined tissues post mortem. The aim of this review is to present a wide range of possible translations of laboratory findings into everyday medical practice. In recent years, because many studies have focused on clinical applications of culture expanded ASCs, we decided to refer to only selected conditions and trials, a list shown in Table 1. The PubMed database was searched for relevant references from January 2013 until April 2020, using the additional species filters “other animals”. Searching criteria included a list of following terms: “adipose-derived stem cell”, “adipose-derived stromal cell”, and “mesenchymal stem cell”. The proposed timeline limitation was implemented to create a review of the most recent publications.

The horse model of tendon lesion has been used to investigate the regenerative potential of ASCs in the treatment of that trauma [65,66,67,68,69]. The surgically induced tendon lesion was treated with injections of cultured ASCs and it was reported that there were not any differences in tendon healing results between horses treated with ASCs and the control group [65,67]. However, some studies have revealed that the single injection of ASCs could promote the organization of collagen fibers, diminish inflammation in injured tissue, stimulate neovascularization, and limit the risk of the progression of tendon lesion in treated horses [66,68,69]. Its immunosuppressive capacities are not yet fully elucidated. Nevertheless, it was noted that an ASCs’ application decreased migration and proliferation of inflammatory cells, promoted the expression of anti-inflammatory cells, and downregulated the synthesis of proinflammatory cytokines, in laboratory models [70,71] The regenerative properties of ASCs have also been examined in rotator cuff repair models [72,73,74,75,76]. It has been noted that the local application of ASCs could result in less pronounced inflammation and increased bone mineral density in histologically evaluated tissues or improved their biomechanical function [72,73,74]. However, others have reported that the subjects treated with ASCs did not benefit from its application as compared with a control group [75,76]. Multiple studies have tried to evaluate the influence of ASC intra-articular injection and intravenous infusion on osteoarthritis treatment outcomes. It has been proven that the administration of ASCs could reduce pain and lameness, suppress the local inflammation, improve mobility and activity, and potentially promote cartilage regeneration [77,78,79,80,81].

To evaluate the efficacy and possible benefits from stem cells application in the management of ischemic injuries, the state of acute ischemia has been provoked in experimental animal models. It was observed that the administration of ASCs could protect tissues against the consequences of acute ischemic injury and modulate their reorganization after myocardial infarction, ischemic stroke, or acute renal ischemic injury [82,83,84,85,86,87,88,89]. Nevertheless, it is worth mentioning that the results of a randomized clinical trial did not support the thesis that the injection of ASCs could minimize kidney injury and could improve renal function after the induced acute ischemia [90].

The application of ASCs is also believed to have a positive impact on chronic wound treatment. It has been established that the administration of ASCs accelerated wound regeneration in animals with induced diabetes [91,92,93,94]. ASCs were found to have the ability to stimulate local angiogenesis, neuroregeneration, collagen deposition, regeneration of the granulation tissue, and suppress periwound inflammation, through autocrine and paracrine mechanisms, which combined could elucidate their application in regenerative medicine [94,95]. ASCs’ wound healing properties were also successfully applied in the treatment of wounds caused by radiotherapy [96].

ASCs were expected to have the ability to increase new bone formation which could be widely applied as a new strategy in reconstructive surgery, however, the obtained results have been ambiguous [97,98,99,100]. The proliferative and osteogenic potential of ASCs was used to investigate their possible utility in dental implant placement immediately after tooth extraction in healthy dogs [101,102]. The most promising outcomes, higher re-osseointegration percentage and increased new bone formation, were noted by Ding et al., in a group of dogs treated with the combination of autologous ASC-derived cell sheets and platelet-rich fibrin membranes transplantation [103]. In contrast, Sánchez-Garcés et al. did not find any significant improvements in bone regeneration after the administration of ASCs [102].

Because of its anti-inflammatory properties, ASC transplantation has appeared to be an effective method for treatment of inflammatory bowel diseases in animal models [103,104,105,106,107,108,109,110]. It was found that ASC administration significantly reduced the secretion of proinflammatory cytokines (tumor necrosis factor-α, interleukin-12, and vascular endothelial growth factor), and improved the clinical disease score and histological parameters in experimentally induced models of murine colitis [103,104,105,108,109,110]. The ASC infusion resulted in a decrease in both clinical inflammatory bowel disease activity index and canine chronic enteropathy clinical activity index, in a group of treated dogs. Clinical remission was noted in nine of 11 dogs [106]. In addition, significant improvement occurred in post-treatment canine inflammatory bowel disease endoscopic index and histological score, however, only four of 11 dogs met the criteria of endoscopic remission. Finally, the full histological remission was not observed in any dog [107]. Moreover, the local application of ASCs was associated with a significantly higher rate of fistula closure in a rat model of perianal fistulas [111].

Some authors have evaluated the potential of ASCs to treat experimentally induced asthma in murine and feline models. Stem cells were isolated from the harvested adipose tissue. Collected samples were subsequently culture expanded. ASCs were administered in intravenous infusions or intratracheally injected. [112,113,114,115,116,117]. It has been found that the application of ASCs reduced lung inflammation, modulated local remodeling, suppressed airway hyperresponsiveness, and as a consequence, improved lung function in mouse models [113,114,115,116,117]. Furthermore, cats treated with ASCs had reduced lung attenuation, bronchial wall thickening scores, and decreased airway hyperresponsiveness as compared with a placebo group. In addition, normalization in eosinophil count was noted only in cats treated with ASCs [112].

Experimental animal studies have been crucial prior to the application of ASCs in humans. A great number of animal models have established the efficacy and safety of stem cell administration in various treatment protocols. Nevertheless, it should be emphasized that those results cannot be directly extrapolated to humans due to interspecies differences. Moreover, it should be said that further studies on the unification of cell preparation protocols are needed. Relatively small study groups are considered to be the main limitations of animal models’ reliability.

### 4.2. Human Clinical Trials

The clinical application of in vitro cultured adipose-derived stem cells seems to be a new promising tool in the treatment of numerous medical conditions. After the appropriate preparation, the surgically obtained specimens of fat tissue are used as a source of ASCs for both autologous and homologous cell transfers and graft implantations. The list of the few recent experimental studies on ASC administration for various diseases in humans is shown in Table 2. The PubMed database was searched for relevant references from January 2013 until April 2020, using the additional species filters “humans”. Searching criteria included a list of following terms: “adipose-derived stem cell”, “adipose-derived stromal cell”, and “mesenchymal stem cell”. The proposed timeline limitation was implemented to create a review of the most recent publications.

In vitro cultured ASCs have been widely used in orthopedics, especially in the process of production of various biomaterials, grafts, and scaffolds [118,119,120,121]. To date, there have been few clinical trials that have focused on using ASCs in standard treatment. According to the results of randomized controlled trials, autologous ASC injections in patients with knee osteoarthritis could efficiently alleviate the pain and were associated with improvement in joint function [122,123,124]. Adipose-derived stem cells have also been used in the process of allograft preparation. The clinical utility of ASC allografts was tested in patients with subtalar joint arthritis as an alternative treatment to typical autologous bone graft in performed subtalar arthrodesis and both methods had similar efficacy in patients’ quality of life enhancement [121,125]. Furthermore, it has been reported that the intradiscal injection of a mixture of ASCs and hyaluronic acid was safe for patients and could significantly reduce chronic discogenic low back pain [126].

The properties of ASCs could also be used in the fields of regenerative and reconstructive medicine. Radiotherapy is commonly administered in patients with head and neck cancers, although the precise dosage treatment often leads to many complications such as salivary gland hypofunction and xerostomia. Grønhøj et al. found that transplantation of previously cultured ASCs could successfully restore the function of submandibular glands as compared with a placebo group [127,128]. Furthermore, stem cells are known for their possible application in chronic wound management. For instance, allogeneic ASC-derived sheets have been used in diabetic foot ulcers treatment. The experimental randomized clinical trial proved their efficiency in wound healing as compared with polyurethane films [129]. Refractory fistulas in patients with inflammatory bowel diseases are considered to be the next example of chronic and hard-to-heal wounds. Is has been proven that injection of autologous or allogeneic ASCs was a safe and efficient method for the treatment of complex perianal fistulas in patients with Crohn’s disease [130,131,132,133]. Autologous ASCs were also used in the surgical treatment of perianal fistulas not associated with inflammatory bowel diseases. Expanded stem cells were injected inside of the fistula tract wall or incubated with the presence of Gore Bio-A fistula plug to adhere to its surface, then, the plug was surgically placed in the fistula tract [132,134,135]. Moreover, it has been reported that ASC injection could promote the replacement of fibrous tissue with new muscles and, as a consequence, contributes to better treatment results in patients with fecal incontinence [136]. ASCs could also be isolated from the buccal fat pad and used in dentistry in the successful treatment of alveolar cleft defects [137]. In addition, its application in craniofacial reconstructive surgery provided a high rate of successful scaffold material integration to surrounding bone with promising long-term observational results [138]. Specific regenerative properties of ASCs could be used in breast reconstructive surgery after a mastectomy has been performed. However, its utility in this condition is quite controversial and could raise concerns about their proliferative effect on residual tumor cells [139,140]. Direct myocardial injection of allogeneic ASCs has been found to be safe and has presented a tendency toward improvement in cardiac function in patients with ischemic heart disease and ischemic heart failure [141]. Saad et al. found that patients with atherosclerotic renovascular disease could benefit from autologous ASC infusion. It has been observed that ASC administration in patients who did not undergo arterial revascularization could increase cortical perfusion, renal blood flow, and decrease renal hypoxia [142].

It has been hypothesized that the administration of ASCs could alter the natural course of progressive neurodegenerative disorders. It is believed that application of this innovative therapy could reduce symptoms, slow the progression of the disease, and delay the occurrence of the disability in many cases [143,144,145,146]. The first step to the implementation of ASCs in multiple system atrophy has already been taken. The safety of intrathecal administration of autologous cells injection has been verified [145]. Similar observations have been noted in patients with secondary-progressive multiple sclerosis. Is has been established that intravenous infusion of ASCs is a safe procedure in that group patients. Nonetheless, there is a lack of sufficient evidence to assess its efficacy [146].

Moreover, it has been demonstrated that autologous stem cell enriched lipotransfer could significantly improve mouth function and subjective psychological measures in patients with systemic sclerosis. To elucidate the mechanism of the anti-fibrotic effect of ASCs, experimental in vitro co-cultures of ASCs and fibroblasts have been performed. ASCs’ paracrine activity reduces the secretion of profibrotic cytokines, modifies the expression of multiple fibrosis associated genes, and finally suppresses the proliferation of fibroblasts [147].

It has been speculated that the application of ASCs could improve the visual parameters in patients with advanced keratoconus after decellularized human corneal lamina transplantation, however, the first trials have shown that the autologous ASC recellularization within corneal stroma did not lead to better treatment outcomes [148,149].

In summary, ASCs still hold great promise in regard to the treatment of numerous medical conditions. The application of either autologous cells or allogeneic grafts could be regarded as a safe procedure that hadwas not been linked to any serious adverse effects for the enrolled patients. The administration of ASCs was found to be an efficient alternative procedure to standard protocols in patients with skeletomuscular diseases and chronic wound management. Nonetheless, its clinical utility in several other conditions is still uncertain and requires further long-term observations.

## 5. Conclusions

In summary, the above information strongly supports the notion that ASCs have a great potential to play a major role in the development of medicine of the 21st century. The methodology used for ASC isolation and culture is well known, with a number of respected regulatory bodies defining minimal criteria of their characterization. At the same time, a number of molecular analyses have implicated them in potential therapies for a number of pathologies. However, because different national regulatory bodies often assume different definitions of the criteria that ASCs need to fulfill to be applied and analyzed in clinical conditions, the procedures required for such research are still far from a worldwide consensus. Nevertheless, there is a range of examples of animal trials and clinical studies employing ASCs, further emphasizing the importance and advancement of studies that could potentially lead to their more widespread use. In addition, in vitro studies will most likely continue to play a significant role in understanding ASC function, both providing the molecular knowledge of their ex vivo properties and possibly serving as an important step in purification and application of those cells in a clinical setting.

## Figures and Tables

**Table 1:** Application of adipose- derived stem cells in animal clinical trials.

Study Title [Reference]	Type of Study	Number of Participants	Medical Condition	Source of ASCs	First Author, (Year)
Effect of single intralesional treatment of surgically induced equine superficial digital flexor tendon core lesions with adipose-derived mesenchymal stromal cells: a controlled experimental trial [65]	Randomized, controlled, blinded experimental study	9 horses	Superficial digital flexor tendon lesion	Autologous	Geburek, (2017)
Equine tendonitis therapy using mesenchymal stem cells and platelet concentrates: a randomized controlled trial [66]	Randomized, controlled trial	8 horses	Superficial digital flexor tendon lesion	Autologous	Carvalho, (2013)
Application of adipose tissue-derived stem cells in a rat rotator cuff repair model [72]	Controlled experimental study	50 rats	Rotator cuff repair model	Allogeneic	Mora, (2014)
Augmentation of chronic rotator cuff healing using adipose-derived stem cell-seeded human tendon-derived hydrogel [73]	Controlled experimental study	53 rats	Chronic rotator cuff repair model	Allogeneic	Kaizawa, (2019)
Evaluation of intravenously delivered allogeneic mesenchymal stem cells for treatment of elbow osteoarthritis in dogs: A pilot study [77]	Open-label clinical trial	13 dogs	Elbow osteoarthritis	Allogeneic	Olsen, (2019)
Synergistic effects of adipose-derived stem cells combined with decellularized myocardial matrix on the treatment of myocardial infarction in rats [85]	Randomized, controlled trial	90 rats	Myocardial infarction	Allogeneic	Qiao, (2019)
Intravenous transplants of human adipose-derived stem cell protect the rat brain from ischemia-induced damage [88]	Randomized, controlled trial	42 rats	Ischemic brain damage	Human ASCs	Gong, (2019)
Efficacy of allogeneic mesenchymal stem cell administration in a model of acute ischemic kidney injury in cats [90]	Randomized, controlled trial	18 cats	Acute ischemic kidney injury	Allogeneic	Rosselli, (2016)
Diabetic human adipose-derived stem cells accelerate pressure ulcer healing by inducing angiogenesis and neurogenesis [95]	Randomized, controlled trial	24 mice	Chronic wound model	Human ASCs	Xiao, (2019)
Therapeutic effects of a recombinant human collagen peptide bioscaffold with human adipose-derived stem cells on impaired wound healing after radiotherapy [96]	Controlled experimental study	12 mice	Radiation-induced skin injury	Human ASCs	Mashiko, (2018)
Bone regeneration of canine peri-implant defects using cell sheets of adipose-serived mesenchymal stem cells and platelet-rich fibrin membranes [101]	Randomized, controlled trial	9 dogs	Dental implant placement	Autologous	Ding, (2019)
Safety and efficacy of allogeneic adipose tissue-derived mesenchymal stem cells for treatment of dogs with inflammatory bowel disease: Clinical and laboratory outcomes [106]	Experimental study	11 dogs	Inflammatory bowel disease	Allogeneic	Pérez-Merino, (2015)
Local application of adipose-derived mesenchymal stem cells supports the healing of fistula: prospective randomised study on rat model of fistulising Crohn’s disease [111]	Prospective, randomized, controlled study	32 rats	Perianal fistula model	Allogeneic	Ryska, (2017)
Intravenous adipose-derived mesenchymal stem cell therapy for the treatment of feline asthma: a pilot study [112]	Controlled experimental study	6 cats	Asthma	Allogeneic	Trzil, (2015)

**Table 2:** Application of adipose—Derived stem cells in human clinical trials.

Study Title [Reference]	Type of Study	Number of Participants	Medical Condition	Source of ASCs	First Author, (Year)
Adipose-derived mesenchymal stem cell therapy in the treatment of knee osteoarthritis: A randomized controlled trial [122]	Randomized, controlled trial	30	Knee osteoarthritis	Autologous	Freitag, (2019)
Subtalar arthrodesis with use of adipose-derived cellular bone matrix compared with autologous bone graft: A multicenter, randomized controlled trial [125]	Prospective, randomized, controlled trial	140	Subtalar joint arthritis	Allogeneic	Myerson, (2019)
Safety and tolerability of intradiscal implantation of combined autologous adipose-derived mesenchymal stem cells and hyaluronic acid in patients with chronic discogenic low back pain: 1-year follow-up of a phase I study [126]	Single-arm phase I clinical trial	10	Chronic discogenic low back pain	Autologous	Kumar, (2017)
Safety and efficacy of mesenchymal stem cells for radiation-induced xerostomia: A randomized, placebo-controlled phase 1/2 trial (MESRIX) [127]	Prospective, randomized, controlled phase 1/2 trial	30	Radiation-induced xerostomia	Autologous	Grønhøj, (2018)
Potential of allogeneic adipose-derived stem cell-hydrogel complex for treating diabetic foot ulcer [129]	Randomized, controlled trial	59	Diabetic foot ulcer	Allogeneic	Moon, (2019)
Expanded allogeneic adipose-derived mesenchymal stem cells (Cx601) for complex perianal fistulas in Crohn’s disease: A phase 3 randomized, double-blind controlled trial [130]	Phase 3 randomized, double-blind controlled trial	212	Complex perianal fistulas in Crohn’s disease	Allogeneic	Panés, (2016)
Early results of a phase I trial using an adipose-derived mesenchymal stem cell-coated fistula plug for the treatment of transsphincteric cryptoglandular fistulas [134]	Phase I clinical trial	15	Transsphincteric cryptoglandular fistulas	Autologous	Dozois, (2019)
Lateral ramus cortical bone plate in alveolar cleft osteoplasty with concomitant use of buccal fat pad-derived cells and autogenous bone: Phase І clinical trial [137]	Phase I clinical trial	10	Alveolar cleft defects	Allogeneic	Khojasteh, (2017)
Cryopreserved off-the-shelf allogeneic adipose-derived stromal cells for therapy in patients with ischemic heart disease and heart failure: A safety study [141]	Phase I clinical trial	10	Ischemic heart disease and heart failure	Allogeneic	Kastrup, (2017)
Autologous mesenchymal stem cells increase cortical perfusion in renovascular disease [142]	Phase 1/2A clinical study	28	Atherosclerotic renovascular disease	Autologous	Saad, (2017)
Intrathecal administration of autologous mesenchymal stem cells in multiple system atrophy [145]	Phase I/II clinical study	24	Multiple system atrophy	Autologous	Singer, (2019)
Adipose-derived mesenchymal stem cells (AdMSC) for the treatment of secondary-progressive multiple sclerosis: A triple blinded, placebo controlled, randomized phase I/II safety and feasibility study [146]	Triple blinded, controlled, randomized phase I/II study	34	Multiple sclerosis	Autologous	Fernández, (2018)
Stem cell enriched lipotransfer reverses the effects of fibrosis in systemic sclerosis [147]	Single-arm experimental study	62	Systemic sclerosis	Autologous	Almadori, (2019)
Corneal stroma enhancement with decellularized stromal laminas with or without stem cell recellularization for advanced keratoconus [148]	Phase I clinical trial	9	Keratoconus	Autologous	Alió del Barrio, (2018)
